# Exceptionally widespread nanomachines composed of type IV pilins: the prokaryotic Swiss Army knives

**DOI:** 10.1093/femsre/fuu001

**Published:** 2014-11-26

**Authors:** Jamie-Lee Berry, Vladimir Pelicic

**Affiliations:** MRC Centre for Molecular Bacteriology and Infection, Imperial College London, London SW7 2AZ, UK

**Keywords:** type IV pilus, type II secretion system, archaellum, class III signal peptide, prepilin peptidase

## Abstract

Prokaryotes have engineered sophisticated surface nanomachines that have allowed them to colonize Earth and thrive even in extreme environments. Filamentous machineries composed of type IV pilins, which are associated with an amazing array of properties ranging from motility to electric conductance, are arguably the most widespread since distinctive proteins dedicated to their biogenesis are found in most known species of prokaryotes. Several decades of investigations, starting with type IV pili and then a variety of related systems both in bacteria and archaea, have outlined common molecular and structural bases for these nanomachines. Using type IV pili as a paradigm, we will highlight in this review common aspects and key biological differences of this group of filamentous structures.

## Abstract (precis)

Using type IV pili as a paradigm, we review common genetic, structural and mechanistic features (many) as well as differences (few) of the exceptionally widespread and functionally versatile prokaryotic nano-machines composed of type IV pilins.

## Graphical abstract

**Figure:**
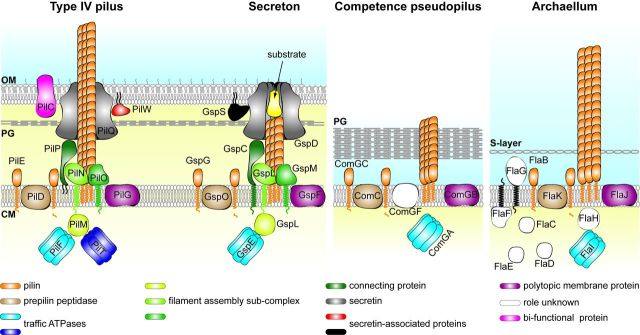
Using type IV pili as a paradigm, we review common genetic, structural and mechanistic features (many) as well as differences (few) of the exceptionally widespread and functionally versatile prokaryotic nano-machines composed of type IV pilins.

## INTRODUCTION

Since the inception of cellular life, prokaryotes (bacteria and archaea) have been faced with a conundrum crucial for their survival: How to efficiently interact with their environment through the formidable barriers that define their very existence? These unicellular organisms have therefore engineered a variety of macromolecular nanomachines on their surface, which are assembled across highly impermeable membrane(s) and/or thick cell walls, and play important and diverse roles in microbial biology. One exceptionally widespread and multipurpose group of nanomachines uses filaments composed of subunits with a characteristic N-terminal sequence motif named class III signal peptide (Szabó *et al.*, 2007), generically named type IV pilins. Because they were the first to be identified and have been extensively studied ever since, the paradigm of this class are the surface-exposed organelles known as type IV pili (Tfp) (Pelicic 2008). Tfp—also known as bundle-forming pili (Bfp) in enteropathogenic *Escherichia coli* (EPEC) (Donnenberg, Zhang and Stone 1997), toxin co-regulated pili (Tcp) in *Vibrio cholerae* (Manning 1997), fimbrial low-molecular-weight protein (Flp) pili in *Aggregatibacter actinomycetemcomitans* (Tomich, Planet and Figurski 2007), etc.—are long surface-exposed filaments composed of type IV pilins, whose biogenesis depends on a set of distinctive proteins. Studies in numerous species of bacteria and archaea have later revealed that several other systems with widely different morphological features are evolutionarily related to Tfp (Hobbs and Mattick 1993; Jarrell, Bayley and Kostyukova 1996) because they are also composed of type IV pilins and assembled by similar sets of proteins. These structures have names as diverse as secreton (Pugsley 1993b), archaellum (Jarrell and Albers 2012) or bindosome (Zolghadr *et al.*, 2007).

In this review, we will provide an overview of the complex biology of the machineries composed of type IV pilins whose biogenesis depends on a conserved set of proteins, for which we would like to introduce the unifying name type IV filaments (Tff). We will briefly list known Tff systems, the wide array of functions they mediate and their astonishing distribution in two out of the three domains of life. With an emphasis on the best characterized Tfp, we will then discuss in detail molecular and structural aspects of Tff and their complex biogenesis, underlining the many important commonalities, as well as a few significant differences.

## TFF: AN AMAZING VARIETY OF MORPHOLOGIES AND ASSOCIATED FUNCTIONS

Unlike any other type of prokaryotic surface nanomachines, Tff come in a variety of shapes and promote a vast array of seemingly unrelated properties such as adhesion, motility, protein secretion and DNA uptake. Hence, these functionally versatile nanomachines can be viewed as the prokaryotic equivalents of the world-famous multitool pocket knife, which has inspired the title of this review. In this section, we will list the different types of Tff and the variety of functions they have been associated with.

### Bacterial Tfp: the Tff paradigm

**Figure 1.**
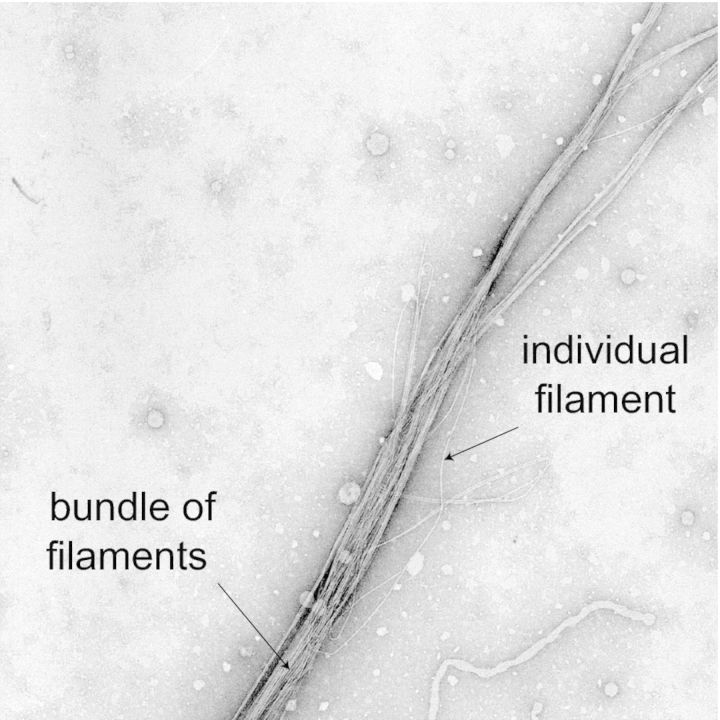
Conspicuous morphological features of bacterial Tfp, i.e. several μm length, 6–8 nm width, flexibility and propensity to interact laterally to form bundles. Bundle of filaments produced by *N. meningitidis* (individual filaments are 60 Å wide) were visualized by EM after negative staining with phosphotungstic acid.

Bacterial Tfp conspicuous morphological features (Fig. 1)—i.e. they are surface-exposed filaments displaying a pronounced flexibility, a propensity to interact laterally to form bundles and are up to 1000 times longer (up to several μm) than they are wide (usually 60–80 Å)—were used in early electron microscopy (EM) studies to define them as a distinct type of pili (Ottow 1975), hence their Tfp moniker. Tfp have since been found and studied in many species of Gram-negative (Mattick 2002; Pelicic 2008) and Gram-positive bacteria (Melville and Craig 2013). Unlike other types of pili, Tfp are capable of retracting and generating forces ranging from 100 pN per single filament (Maier *et al.*, 2002), to several nN for bundles (Biais *et al.*, 2008). Retraction, which occurs through rapid depolymerization of pilin subunits, has so far been directly demonstrated only for one sub-class of Tfp (Tfpa see below). This is consistent with the fact that the PilT ATPase powering this process (Merz, So and Sheetz 2000) is restricted to the corresponding bacterial species. It remains a burning question whether other sub-classes of Tfp and/or other Tff can also retract as there is at best only indirect evidence so far (Bieber *et al.*, 1998; Zahavi *et al.*, 2011). This huge force generation makes Tfp the most potent linear molecular motor described to date. Critically, it endows Tfp with properties not commonly associated with other pili and can even influence their morphology, i.e. when under tension *Neisseria gonorrhoeae* Tfp reversibly transition into a 40% narrower elongated conformation (Biais *et al.*, 2010).

#### Tfp are ‘sticky’ organelles

Like other types of bacterial pili, Tfp mediate attachment to and colonization of a wide variety surfaces, both abiotic (plastic, glass, metal, etc.) and biotic (host cells and extracellular matrix in commensals and pathogens). Attachment to biotic surfaces makes Tfp key virulence factors in several human pathogens responsible for infections leading to dramatic morbidity and/or mortality worldwide—cholera, diarrhoea, meningitis and gonorrhoea, to cite but a few—and is the main reason Tfp have been a hot topic for research for decades. Tfp mediate adhesion in several ways in contrast to other types of pili that most often harbour a minor (low-abundance) subunit with intrinsic adhesive properties at their tip, e.g. type I pili (Lillington, Geibel and Waksman 2014). For example, the major Tfp subunit itself can have adhesive properties, such as the Bfp subunit that is a lectin with affinity for *N*-acetyllactosamine receptors on human host cells (Hyland *et al.*, 2008), or PilA1 that mediates adhesion of the Gram-positive *Ruminococcus albus* to cellulose in the gastrointestinal tract of ruminants (Pegden *et al.*, 1998; Rakotoarivonina *et al.*, 2002). Alternatively, minor pilins can play important roles in attachment, such as PilV in the pathogenic *Neisseria* species *N. meningitidis* and *N. gonorrhoeae* (Winther-Larsen *et al.*, 2001; Brown *et al.*, 2010). PilV first binds CD147, mediating tight adhesion to host cells (Bernard *et al.*, 2014), and then it binds β-adrenergic receptors (Coureuil *et al.*, 2010) triggering the formation of membrane protrusions around adhering meningococci and enhancing their ability to resist mechanical forces generated by the blood flow (Mikaty *et al.*, 2009). Non-pilin proteins associated with Tfp can also play key roles in adhesion as shown for PilC/PilY1 in multiple species (Rudel *et al.*, 1992; Nassif *et al.*, 1994; Kehl-Fie, Miller and St Geme 2008; Heiniger *et al.*, 2010). Recently, an Arg-Gly-Asp motif in the N-terminal domain (NTD) of *Pseudomonas aeruginosa* PilY1 was found to bind integrin (Johnson *et al.*, 2011), providing evidence that it is a *bona fide* adhesin. Since the NTD of PilC/PilY1 is species-specific, it is likely that orthologues in different species mediate adhesion to different receptors. Finally, Tfp retraction can influence their adhesive properties. For example, *N. gonorrhoeae* promotes colonization by mechanically stimulating pathways in host cells upon pilus retraction (Howie, Glogauer and So 2005).

#### Tfp promote interbacterial contacts

Another very common property of Tfp that influences attachment to surfaces is their ability to promote interaction between neighbouring bacteria via pilus–pilus contacts. These contacts lead to the formation of aggregates or micro-colonies (Kirn *et al.*, 2000), which can even become biofilms if embedded within a matrix of extracellular polymeric substance (O’Toole and Kolter 1998). Although they both involve pilus–pilus contacts, it should be pointed out that formation of micro-colonies and pilus bundling are distinct properties (Kirn *et al.*, 2000). Formation of micro-colonies can be promoted by the major pilus subunit as in *V. cholerae* Tcp (Chiang *et al.*, 1995), or by a minor pilin such as PilX in *N. meningitidis* (Helaine *et al.*, 2005). PilX subunits in the filaments of interacting meningococci are thought to brace against each other upon pilus retraction through surface-exposed ‘hooks’ and thereby stabilize micro-colonies in face of pilus retraction (Helaine *et al.*, 2007), although an alternative hypothesis has recently been proposed (Imhaus and Dumenil 2014). The formation of micro-colonies, which can also be seen in liquid culture, is however not limited to enhancing surface colonization. As shown for the R64 plasmid thin pilus of *E. coli* (Yoshida *et al.*, 1998), Tfp-mediated aggregation promotes subsequent exchange of DNA between cells.

#### Tfp power twitching motility

Almost all bacterial species with retractile Tfp undergo surface-associated motility known as twitching motility (or social motility in *Myxococcus xanthus*) because cells exhibit jerky movements (Henrichsen 1972). Bacteria use Tfp as ‘grappling hooks’ and upon PilT-mediated pilus retraction pull themselves towards the site where the pilus is attached (Mattick 2002). The force generated by a single filament retraction (Maier *et al.*, 2002) allows the bacterium to move 10 000 times its own body weight (Baker, Biais and Tama 2013), which results in rapid movement (Merz, So and Sheetz 2000). The irregular motion, abrupt turns and changes of direction, characteristic of twitching, are due to the release of single filaments, while others are still under tension, so that the bacterium rapidly ‘slingshots’ to a new orientation (Jin *et al.*, 2011). Twitching motility has probably evolved to allow surface exploration that can be random or directed, e.g. towards light sources in the cyanobacterium *Synechocystis* sp. (Bhaya *et al.*, 2000), and can even be optimized for 2D exploration when bacteria stand upright and ‘walk’ (Gibiansky *et al.*, 2010).

#### Tfp promote DNA uptake during natural transformation

In contrast to the widespread nature of twitching motility, only a subset of bacterial species with retractile Tfp use these to promote the earliest step of natural transformation, i.e. import (or uptake) of free extracellular DNA across the outer membrane and/or thick layer of peptidoglycan (PG) (Chen and Dubnau 2004). In these species, DNA uptake is directly powered by pilus retraction and is abolished in a *pilT* mutant (Wolfgang *et al.*, 1998a). Imported DNA is used to generate genetic diversity, as a template for the repair of damaged DNA, or as a source of food (Chen and Dubnau 2004). Tfp bind extracellular DNA (van Schaik *et al.*, 2005) most likely through a major or minor pilin, as confirmed by the recent discovery that the ComP minor pilin in the Neisseriaceae family has intrinsic DNA-binding ability (Berry *et al.*, 2013; Cehovin *et al.*, 2013). Furthermore, ComP binds better to short and specific sequence motifs hyperabundant in these species genomes, explaining how they manage to preferentially take up their own DNA.

#### Uncommon and/or indirect Tfp properties

Another property of bacterial Tfp, which further extends the versatility of this class of filaments, might be viewed as ‘exotic’ or as a mere curiosity. In *Geobacter* species, Tfp have been found to be electrically conductive ‘nanowires’ transferring electrons from the cells to extracellular electron acceptors (Reguera *et al.*, 2005). Although it has been proposed that conductivity might result from electrons ‘hopping’ between cytochromes attached to *Geobacter* Tfp (Boesen and Nielsen 2013), filaments are likely to have intrinsic metal-like electron-conductive properties through stacking of aromatic residues of the major pilin PilA. Accordingly, filaments composed of *pilA* mutants lacking these residues have reduced conductive properties (Vargas *et al.*, 2013).

It is worth mentioning here that retractile Tfp have also been hijacked by bacterial viruses. Some bacteriophages bind to the side or tip of Tfp, and are brought in contact with a cell surface-associated receptor upon pilus retraction (Skerker and Shapiro 2000). Historically, this is an important property since it is the observation by EM that shortening of *P. aeruginosa* pili occurred after phage attachment and was necessary for phage infection that led Bradley to propose that Tfp are capable of retraction (Bradley 1972). Furthermore, the finding that phage-resistant mutants that were unable to retract their pili were also defective for twitching allowed the same author to link Tfp retraction with twitching motility for the first time (Bradley 1980).

### Other widespread bacterial Tff

#### Competence (pseudo)pili

In most naturally competent bacterial species, DNA uptake is not mediated by Tfp but rather by elusive Tff structures known as competence ‘pseudopili’ formed of a major ‘pseudopilin’, which cannot be directly visualized by EM because they are too short (Chen and Dubnau 2004). Key evidence for their existence is the recent discovery of extended competence organelles in *Streptococcus pneumoniae* (Laurenceau *et al.*, 2013; Balaban *et al.*, 2014), which remained unnoticed for decades in this species where competence has been intensively investigated (Johnston *et al.*, 2014). Although no PilT is associated with competence pseudopili, a study in *Bacillus subtilis* showed that these are nevertheless force-generating motors exerting forces in excess of 40 pN on free DNA, and transporting it into the cell in a linear fashion (Maier *et al.*, 2004). Transport is powered by proton motive force but the underlying molecular mechanism for force generation remains to be determined.

#### Type II secretion systems

Rather than for DNA import, pseudopili are used by many Gram-negative species as part of the secreton machinery to ‘push’ or ‘lift’ fully folded periplasmic proteins or protein complexes into the extracellular milieu across a dedicated channel in the outer membrane (Douzi, Filloux and Voulhoux 2012; Korotkov, Sandkvist and Hol 2012; Nivaskumar and Francetic 2014). Perhaps misleadingly, this process is known as type II secretion, whereas type IV secretion is mediated by a machinery unrelated to Tfp (Low *et al.*, 2014). However, these unfortunate differences in nomenclature predate the discovery that type II secretion systems (T2SS) are evolutionarily related to Tfp, and further support our proposal of a unifying Tff name. As for competence pseudopili, T2SS pseudopili have never been directly visualized, probably because they are too short, barely spanning the periplasmic space (Douzi, Filloux and Voulhoux 2012; Nivaskumar and Francetic 2014). However, a key piece of evidence for their existence is the fact that surface-exposed filaments similar to Tfp, named ‘hyper-pseudopili’, can be seen when T2SS-harbouring bacteria are genetically engineered to overexpress the major pseudopilin (Sauvonnet *et al.*, 2000; Durand *et al.*, 2003; Vignon *et al.*, 2003). T2SS-exported proteins, often enzymes, play important roles in lifestyles of the secreting bacteria by providing them with essential nutrients or by having toxic effects on host cells in pathogens (Nivaskumar and Francetic 2014). Interestingly, protein secretion can also be mediated by some Tfp machineries as in *V. cholerae* and *Dichelobacter nodosus* (Kirn, Bose and Taylor 2003; Han *et al.*, 2007). This blurs the lines between the different Tff and strengthens the notion that they are a homogeneous class of nanomachines.

### Archaeal Tff

It is now clear that Tff are also widespread in a second domain of life, archaea. Although they often have different morphological features from bacterial Tfp—notably diameters up to 140 Å (Jarrell *et al.*, 2013)—*bona fide* Tfp (long surface-exposed organelles composed of type IV pilins whose biogenesis depends on similar sets of proteins) have been identified in many archaeal species. Two main groups of archaeal filaments are distinguished based on the functions they have been associated with. Those filaments involved in attachment to surface and/or promotion of interarchaeal contacts are generically called pili. As in bacteria, Tfp-mediated aggregation can promote subsequent exchange of DNA between archaeal cells as shown for UV-induced pili of *Sulfolobus* (Fröls *et al.*, 2008). Archaeal filaments that power swimming such as in *Sulfolobus acidocaldarius* are defined as flagella. Indeed, these Tff drive swimming by rotating (Shahapure *et al.*, 2014) and are thus functional analogues of bacterial flagella (Jarrell *et al.*, 2013). To underline these differences, a new name ‘archaellum’ has been coined for these archaeal flagella (Jarrell and Albers 2012), but it is not universally accepted. Intriguingly, it has been shown that the FlaI ATPase powers both archaellum assembly and rotation (Reindl *et al.*, 2013). The fact that a protein universally energizing Tff assembly (see below) can power filament rotation in archaea might have biological implications for this whole class of filaments, as will be discussed later. Among the many reasons why archaella might be the only Tff powering swimming are a higher rigidity, the possibility that they are Archimedes’ ‘screws’, the presence of additional machinery components, etc.

Possibly the most peculiar Tff machinery is the elusive *S. solfataricus* structure named bindosome that facilitates sugar uptake, which consists of several surface-exposed sugar-binding proteins that harbour class III signal peptides (Zolghadr *et al.*, 2007). The bindosome has never been visualized and evidence that it produces (or not) even short filaments is still to be obtained. However, the bindosome surface localization depends on a canonical Tff named Bas.

## TFF BIOGENESIS RELIES ON A LARGE AND CONSERVED SET OF DEDICATED PROTEINS

**Figure 2.**
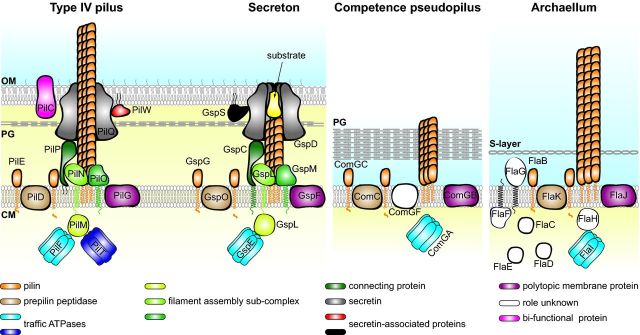
Schematic representation of different Tff nanomachines: Tfp, T2SS, competence pseudopili and archaella. For Tfp, the well-characterized Tfpa system in *N. meningitidis* is shown. It should be noted that the protein nomenclature varies widely in other Tfpa-expressing species, although all the proteins are highly conserved. However, a major difference in Gram-positive piliated species is the absence of the proteins forming the outer membrane sub-complex (PilC, PilP, PilQ and PilW). For T2SS, the system exporting pullulanase in *K. oxytoca* is shown, but the proteins have been given their unifying Gsp names. For competence pseudopili, *B. subtilis* has been chosen. For archaeal flagella, we have chosen the representative *M. maripaludis* archaellum. All these systems are evolutionarily related as they are composed of proteins that show sequence and/or structural similarity and perform the same functions. To facilitate comparisons, proteins of similar function have identical colour. In brief, major (pseudo)pilins are processed by a dedicated prepilin peptidase, which removes a short hydrophilic leader peptide. For the sake of clarity, minor (pseudo)pilins that also undergo this processing are not shown. Traffic ATPases power filament extension from the inner membrane through ATP hydrolysis. The PilT ATPase which powers filament retraction has so far been identified only in Tfpa. The energy generated by ATP hydrolysis is translocated across the membrane by a multiprotein sub-complex, although the polytopic protein showing universal sequence conservation (purple) has also been proposed to play this role. In Gram-negative species, the inner membrane sub-complex is linked via a connecting protein to an outer membrane sub-complex centred on a multimeric channel known as the secretin. Several other proteins important for secretin stability/function are also part of this sub-complex. To facilitate visualization, the secretin dodecamer is shown as a vertical cross-section. OM, outer membrane; PG, peptidoglycan; CM, cytoplasmic membrane.

Tff are not only formed of subunits sharing a well-defined and conserved N-terminal motif but their biogenesis relies on conserved multiprotein machineries (Fig. 2), which is in support of their evolutionary relationship.

### Type IV pilins

**Figure 3.**
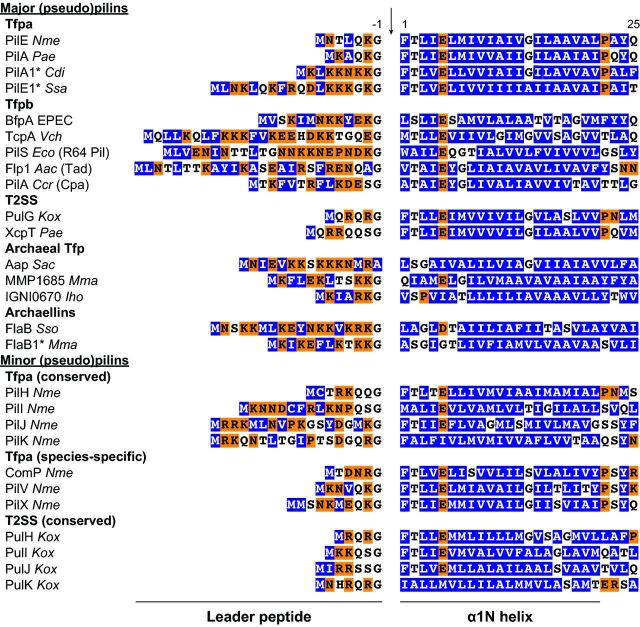
Conserved N-terminal sequence motif defining pilin subunits in a variety of Tff. This motif, known as class III signal peptide (Szabó *et al.*, 2007), is composed of a hydrophilic leader peptide followed by a stretch of hydrophobic residues (except for a negatively charged Glu_5_). The 6–26 aa leader peptide contains a majority of hydrophilic (shaded in orange) and neutral (no shading) residues, and invariably ends with a Gly (except in archaea). The following tract of 21 predominantly hydrophobic residues (shaded in blue) forms an extended α-helix that is the main assembly interface of subunits within Tff. This class III signal peptide is recognized by a dedicated prepilin peptidase and cleaved (indicated by a vertical arrow) after the conserved Gly_-1_. *Filaments in these species might be composed of more than one major pilin. *Nme*, *N. meningitidis*; *Pae*, *P. aeruginosa*; *Cdi*, *C. difficile*; *Ssa*, *Streptococcus sanguinis*; *Vch*, *V. cholerae*; *Eco*, *E. coli*; *Aac*, *A. actinomycetemcomitans*; *Ccr*, *C. crescentus*; *Kox*, *K. oxytoca*; *Sac*, *S. acidocaldarius*; *Mma*, *M. maripaludis*; *Iho*, *Ignicoccus hospitalis*; *Sso*, *S. solfataricus*.

Tff subunits, which are synthesized as precursors, share a N-terminal class III signal peptide (Fig. 3) that is well defined and distinctive (Dalrymple and Mattick 1987; Szabó *et al.*, 2007). These subunits will be generically referred to as type IV pilins, although this might be a misnomer because not all of them are involved in the biogenesis of pili. The class III signal peptide starts with a leader peptide containing a majority of hydrophilic and neutral residues, whose length varies between 6 and 26 amino acids (aa) in *P. aeruginosa* PilA (Paranchych *et al.*, 1979) and *A. actinomycetemcomitans* Flp1 (Kachlany *et al.*, 2001), respectively. Although there are many exceptions in archaea, this leader peptide invariably ends with a conserved Gly, and is followed by a tract of 21 predominantly hydrophobic residues (Fig. 3). A negatively charged Glu_5_ residue almost invariably interrupts this stretch, although it is often absent in archaeal pilins (Szabó *et al.*, 2007; Jarrell *et al.*, 2013). Importantly, these features distinguish class III signal peptides, which are processed by a dedicated prepilin peptidase after the conserved Gly on the cytoplasmic side of the membrane (Nunn and Lory 1991) (Fig. 2), from standard (class I) and lipoprotein (class II) signal peptides, which are processed by different peptidases on the periplasmic side (Pugsley 1993b).

Historically, differences in length of prepilins and their leader peptides in the extensively studied Tfp systems were used to define two sub-classes of Tfp, Tfpa and Tfpb (Giltner, Nguyen and Burrows 2012). Prepilins of Tfpa are shorter (140–170 aa) with shorter leader peptides (less than 10 residues), while Tfpb prepilins are longer (180–200 aa) with longer leader peptides (about 15–30 aa) (Fig. 3). This distinction is consistent with important differences between the machineries involved in their biogenesis (Pelicic 2008). The discovery and study of many other Tff in more varied species means that these traditional length parameters are not valid anymore. Nevertheless, phylogenetic analysis of bacterial prepilins showed that two families consistent with the original classification can be distinguished (Kachlany *et al.*, 2001). Type IVa prepilins, some of which have leader peptides as long as 18 residues, share substantial sequence homology in their N-terminal hydrophobic stretch (Fig. 3) and they are by far the most widespread, being found both in pili and pseudopili (Giltner, Nguyen and Burrows 2012). Type IVb prepilins, which can be as short as 44 residues in *Caulobacter crescentus* (Skerker and Shapiro 2000), also group together although they show less sequence homology, except for the Flp sub-family (Tomich, Planet and Figurski 2007). Most archaeal prepilins do not readily fit in the above two families and are likely to form one or more families of their own (Desmond, Brochier-Armanet and Gribaldo 2007; Szabó *et al.*, 2007), which could tentatively be named type IVc, type IVd, etc.

### Tff biogenesis machineries

The complete sets of proteins dedicated to Tff biology have been defined by systematic genetic studies in several model systems: Tfpa in *P. aeruginosa* and *N. meningitidis* (Alm and Mattick 1997; Carbonnelle *et al.*, 2006), Tfpb in EPEC, *V. cholerae* and *E. coli* (Yoshida, Kim and Komano 1999; Kirn, Bose and Taylor 2003) and T2SS in multiple species such as *Klebsiella oxytoca*, *Erwinia chrysanthemi* and *P. aeruginosa* (Douzi, Filloux and Voulhoux 2012; Nivaskumar and Francetic 2014). This revealed that although Tff are primarily polymers of a single protein, their biogenesis requires complex machineries of 10–18 proteins. In most systems—Tfpb, T2SS, archaeal Tff—the corresponding genes cluster together but they are scattered throughout the genome for Tfpa, except in the few Gram-positive species in which these are found (Pelicic 2008; Douzi, Filloux and Voulhoux 2012; Jarrell and Albers 2012; Melville and Craig 2013; Nivaskumar and Francetic 2014). Unlike for Tfpb that are more heterogeneous—Flp that are assembled by the conserved tight adherence (Tad) locus are an exception (Tomich, Planet and Figurski 2007)—genes are conserved ‘en bloc’ for Tfpa or T2SS with similar genetic organizations. In Tfpa for example, there are virtually no differences between *P. aeruginosa* and *N. meningitidis* that are distant γ and β Proteobacteria. The only major difference might be the *pilY2* gene essential for piliation, which is restricted to *P. aeruginosa* (Alm *et al.*, 1996b). It should be noted here that additional ‘accessory’ proteins are often key for Tff function(s) while being dispensable for filament biogenesis, which makes Tff biology even more complex. For example, in *N. meningitidis*, there are seven accessory proteins—three of which (PilT, PilU and PilZ) are widely conserved—that modulate one or several Tfpa functions (Brown *et al.*, 2010): three minor pilins (ComP, PilV and PilX), three ATPases (PilT, PilT2 and PilU) and PilZ.

The most important finding in the above studies was that Tff are unequivocally a homogeneous class of filaments since many proteins involved in their biogenesis show sequence homology, several of which are even universally conserved (Fig. 2). These conserved proteins are often designated ‘core’ but recent structural findings (see below) show that this distinction is misleading and should probably be abandoned. This set of conserved proteins consists of at least one protein (but more often several) with a class III signal peptide, a dedicated prepilin peptidase for prepilin processing, a ‘traffic’ ATPase to power filament assembly and a polytopic cytoplasmic membrane protein of unknown function. Intriguingly, many of the other non-core proteins important for Tff biogenesis exhibit no obvious sequence conservation between the different systems, which led to questioning whether a unique mechanism was used for Tff biogenesis (Pelicic 2008). This is nevertheless likely to be the case as suggested by recent structural data showing that non-core proteins exhibiting no sequence homology have strikingly similar 3D structures. For example, the structure of the N-terminal part of BfpC that is involved in Tfpb biogenesis (Yamagata *et al.*, 2012) is similar to the structures of PilM involved in Tfpa biogenesis (Karuppiah and Derrick 2011) and GspL from T2SS (Abendroth *et al.*, 2004a). Unfortunately, although a unified Gsp (general secretory pathway) nomenclature has been proposed for T2SS (Pugsley 1993b), these proteins have different names in different species or systems, which makes comparisons difficult. To help the reader, proteins performing the same function in different systems have the same colour in Fig. 2. It should also be noted that, unless stated otherwise, the *N. meningitidis* nomenclature will be privileged in the rest of this review.

## TFF ARE VIRTUALLY UNIVERSAL IN PROKARYOTES

**Figure 4.**
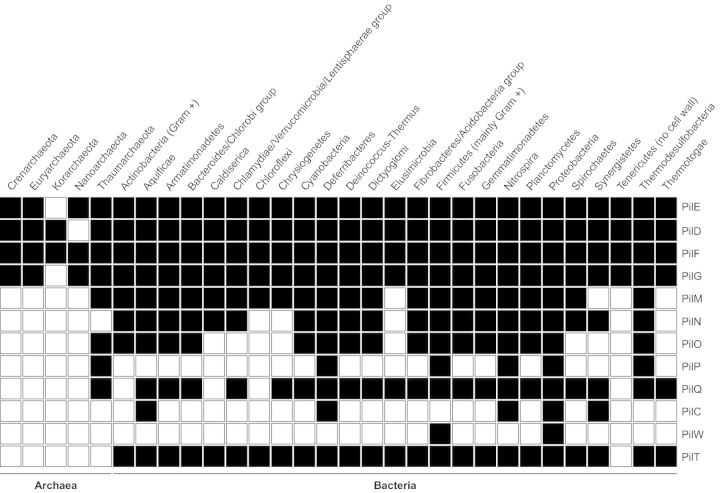
Phylogenetic distribution of the proteins involved in Tff biogenesis in archaea and bacteria. Using BioMart (Guberman *et al.*, 2011), we have performed a global search of the InterPro database (Hunter *et al.*, 2012) for signature motifs found only in proteins dedicated to Tff biogenesis. A black box indicates that the corresponding protein is found in the analysed phylum, while a white box indicates that it is absent. PilE, IPR007047, IPR002774 or IPR012912; PilD, IPR010627 or IPR000045; PilF, IPR007831 or IPR001482; PilG, IPR018076; PilM, IPR005883; PilN, IPR007813; PilO, IPR007445; PilP, IPR007446; PilQ, IPR001775 or IPR013355; PilC, IPR008707; PilW, IPR013360; PilT, IPR006321. In Firmicutes, the outer membrane sub-complex proteins (PilP, PilQ and PilW) are found only in the very few Gram-negative species in this phylum.

Various types of Tff nanomachines have been identified and experimentally studied in many prokaryotic species. However, their global distribution remains an open question. This can be addressed by mining the ever increasing amount of sequence data in the databanks in search of signature motifs found uniquely in proteins dedicated to Tff biogenesis. Using BioMart (Guberman *et al.*, 2011), we have therefore performed a global search of the InterPro database (Hunter *et al.*, 2012) for the distribution of such motifs across all the species sequenced so far. This analysis shows that Tff are one order of magnitude more widespread than previously anticipated (Pelicic, 2008), and are actually virtually universal in prokaryotes (Fig. 4). This is likely to be a consequence of their extreme functional versatility and their ancient nature since a Tff, whose function can only be guessed, was already present in a common ancestor to bacteria and archaea that have diverged more than 3 billion years ago.

The most obvious sequence signature to start this bioinformatic search was the one found in Tff subunits: the distinctive class III signal peptide motif (Fig. 3) (Dalrymple and Mattick, 1987; Szabó *et al.*, 2007). A simple query using InterPro domains absolutely specific for bacterial (IPR012912 and/or IPR007047) and archaeal type IV prepilins (IPR002774) suggests that Tff machineries are encoded in the genomes of approx. 1800 different species. These species span 4/5 phyla of archaea (Korarchaeota are apparently the only exception) and all 26 phyla or group of phyla of bacteria (Fig. 4). Strikingly, this estimate is even likely to be conservative since simple queries using domains specific for other components invariably found in Tff machineries, namely the prepilin peptidase (IPR010627 and/or IPR000045), the traffic ATPase (IPR007831 and/or IPR001482) and the PilG polytopic membrane protein (IPR018076), each returned more than 2000 different species, including some in which no prepilins could be found. The possibility that prepilins not readily identified using the above pilin signatures might be present in those ‘extra’ species is supported by the PilFind algorithm that predicts many non-canonical type IV prepilins (Imam *et al.*, 2011), including a new class that we are currently characterizing (Pelicic, unpublished). This new class of prepilins corresponds to a well-defined domain of so far unknown function that is widespread in Actinobacteria (275 different species).

These findings are strengthened by a multiquery using all the above domains. This search revealed that the genomes of 1656 different species, in which type IV prepilins are found, encode simultaneously a prepilin peptidase, a traffic ATPase and the polytopic cytoplasmic membrane protein (Fig. 4). These species span all the phyla of archaea and bacteria listed above, except Nanoarchaeota in which no prepilin peptidase could be detected. Several other interesting observations arose from these queries. Firstly, prepilin peptidases often consist of two domains, one of which (IPR000045) always corresponds to the peptidase domain. In bacteria, the most common second domain (IPR010627) is almost certainly responsible for the well-known N-methylation of the first residue of many mature pilins (Strom *et al.*, 1993). In archaea, where this N-methylation is not observed (Jarrell *et al.*, 2013), IPR010627 is replaced by IPR009655 whose function is unknown. This suggests that archaeal pilins might undergo another sort of N-terminal modification. Interestingly, in *Methanococcus maripaludis* Tfp, the N-terminal Gln of the major pilin is modified into a pyroglutamate (Ng *et al.*, 2011). Secondly, almost 1000 species encode the above four proteins as well as PilT retraction ATPase (IPR006321), and are likely to express Tff capable of retraction. Of these 1000 species, 500 also encode the PilM, PilN, PilO proteins (IPR005883, IPR007813 and IPR007445, repectively) and are likely to express *bona fide* Tfpa (Pelicic, 2008). Strikingly, the entire set of 15 proteins involved in Tfpa biogenesis in *N. meningitidis* is found in as many as 270 different species of Proteobacteria.

## TFF: COMMON STRUCTURAL ASPECTS

**Figure 5:**
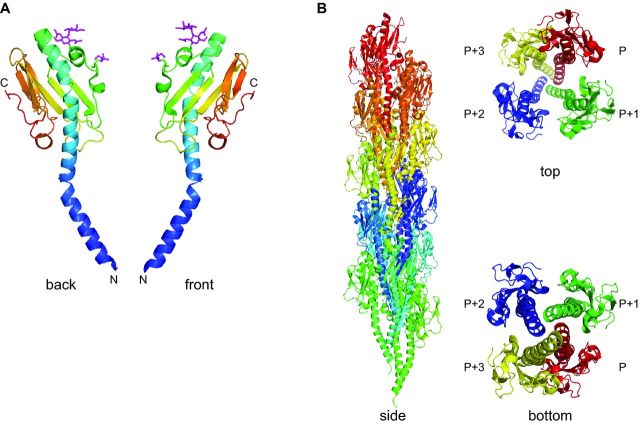
Common structural features of Tff and their subunits illustrated with *N. gonorrhoeae* Tfpa (Craig *et al.*, 2006). **(A)** Structure of the full-length PilE from *N. gonorrhoeae* (PDB entry 2HI2) showing the conserved lollipop shape with a protruding α1N-helix (back view on the left, front view on the right). Post-translational modifications on the αβ-loop, carbohydrate at Ser_63_ and phosphate at Ser_68_, are shown in magenta. **(B**) Structural model of gonococcal Tfp (PDB entry 2HIL) obtained by combining X-ray crystallography and cryo-EM (side view on the left, top view on the upper right, bottom view on the lower right). In the 60 Å diameter right-handed 1-start helical assembly, the pilins are arranged in an ‘ear of wheat’ fashion (side view). The α1N-helices provide the main polymerization interface and are buried within the filament core parallel to its long axis (top and bottom views). Figures were generated using PyMOL (http://www.pymol.org).

Tff have been intensively studied from a structural point of view. This has confirmed that they are a homogeneous group of filamentous nanomachines in which pilin subunits share a distinctive fold and are assembled in a similar way (Fig. 5).

### Type IV pilins: a universally conserved structural fold

Once their leader peptide is processed, Tff subunits share an N-terminal tract of highly hydrophobic residues with predicted propensity to form an extended α-helix. There is little, if any, sequence homology in the rest of these proteins. Notwithstanding, structural studies have revealed that type IV pilins share a common 3D architecture (Craig, Pique and Tainer 2004). Full-length structures of mature Tfp-forming type IVa pilins from *N. gonorrhoeae* (Parge *et al.*, 1995), *P. aeruginosa* (Craig *et al.*, 2003) and *D. nodosus* (Hartung *et al.*, 2011), all resemble ‘lollipops’, with a ‘stick’ and a globular head (Fig. 5A). The stick corresponds to the N-terminus of the pilins and consists of a ∼50 residue α-helix (α1). The N-terminal half of α1 (α1N) protrudes from the C-terminal globular head and corresponds primarily to the stretch of 21 highly hydrophobic residues that is part of the class III signal peptide (Fig. 3). The C-terminal half of α1 (α1C) is packed against the globular head that consists mainly of a β-meander motif of four antiparallel β-strands. Most of the structural diversity between pilins lies in the two regions flanking the β-meander, the αβ-loop that connects α1 and the first β-strand of the β-meander, and the C-terminus known as the D-region because it is usually stapled to the last β-strand of the β-meander by a disulphide bond. D-region is, however, a misnomer since these C-terminal Cys are not always conserved and the disulphide bond can be replaced by a network of hydrogen bonds as in the *D. nodosus* pilin (Hartung *et al.*, 2011). Recently, the full-length structure of a much shorter (66 aa) type IVa pilin subunit of *Geobacter sulfurreducens* nanowires has been determined in detergent micelles (Reardon and Mueller 2013). This structure, which almost exclusively consists of α1 (i.e. there is no globular head), confirmed that the first 21 residues are inserted within the membrane prior filament assembly and showed that an extended N-terminal α-helix might be viewed as the minimal common feature of type IV pilins.

The many other structures available for type IVa and type IVb major and minor (pseudo)pilins lack the protruding and highly hydrophobic α1N—for a review see Giltner, Nguyen and Burrows (2012). Truncation makes the recombinant proteins soluble and more readily amenable to purification and structural characterization. It has (i) minimal structural impact as shown with the *P. aeruginosa* PAK pilin for which full-length and truncated structures are essentially identical (Craig *et al.*, 2003), and (ii) no consequence on the utility of these structures since the missing hydrophobic region can reliably be modelled as an extended α-helix. All these structures confirm that the overall architecture of type IV pilins is conserved, i.e. α1C packs against a β-meander motif that is composed of three to seven antiparallel β-strands depending on the size of the protein. As mentioned above, the structural diversity, which accounts for the functional differences between corresponding proteins and/or filaments, lies in the αβ-loop and D-region. For example, the D-region of the minor pilin PilX that is key for the formation of bacterial aggregates in *N. meningitidis* consists of a short ‘pigtail’ α-helix and a hook that protrudes from the surface of the filaments (Helaine *et al.*, 2007). Interestingly, major pilins undergo post-translational modifications in these regions (Fig. 5A), most often addition of glycans, although phosphorylcholine, phosphoethanolamine and phosphoglycerol, have also been described (Forest *et al.*, 1999; Hegge *et al.*, 2004; Gault *et al.*, 2013). The functional significance of these modifications is unclear and varies from system to system. In bacteria, they are usually dispensable for Tfp assembly and have little effect on Tfp-mediated functions (Marceau *et al.*, 1998; Smedley *et al.*, 2005), while in archaea non-glycosylated subunits cannot be assembled in filaments (Chaban *et al.*, 2006; VanDyke *et al.*, 2008).

Until very recently, only structures of pilins from Gram-negative bacteria were available and it could only be speculated that the above architecture was universally conserved. However, the recently determined structure of the minor pilin PilJ from the Gram-positive *Clostridium difficile* (Piepenbrink *et al.*, 2014) not only displays the typical pilin fold but is, curiously, the only type IV pilin with a dual pilin fold likely to have resulted from a duplication or a fusion event (Piepenbrink *et al.*, 2014). This finding is important because it strengthens the notion that the classical fold is likely to be universal in type IV pilins. However, it would be interesting to determine the structures of more distant members, notably pilins from archaea (Szabó *et al.*, 2007) which display important sequence differences (Fig. 3) or the very short pilins from the Flp sub-family (Kachlany *et al.*, 2001). The latter ones are puzzling because they are even shorter than the *G. sulfurreducens* pilin (Reardon and Mueller 2013) and possibly consist only of the α1N-helix, which might thus be viewed as the minimal universally conserved structural fold defining type IV pilins.

### Similar Tff models

Consistent with this universally conserved fold, all models predicting how type IV pilins are arranged within filaments agree that Tff are helical polymers in which α1N-helices provide the principal polymerization interface and are buried within the filament core parallel to the filament axis, in an ‘ear of wheat’ fashion (Fig. 5B). This is true even in archaeal species producing ‘unorthodox’ Tff (Wang *et al.*, 2008; Yu *et al.*, 2012). Several different helical models have been proposed using a variety of methods. The prototype model for Tfpa, which has been determined for *N. gonorrhoeae* filaments (Craig *et al.*, 2006), is marked by high ridges (corresponding to the αβ-loop and D-region) and deep grooves that wind around the filament axis (Fig. 5B). The α1-helices are within the core of the filament, almost parallel to its long axis. In the 60 Å diameter right-handed 1-start helix, consecutive subunits are separated by a rise of 10.5 Å and show 101º azimuthal rotation. In this pseudo-atomic resolution model, charges in α1 are neutralized by inter-subunit salt bridges, such as the one between Glu_5_ of one protomer (P) and the N-terminal amide of Phe_1_ of the next (P+1) (Craig *et al.*, 2006). A 3-start mechanism for Tfpa assembly was proposed (Craig *et al.*, 2006), in which three pilin subunits are added simultaneously around the filament circumference.

Although the corresponding pilin subunit and the filament are significantly larger, a similar approach (which was refined by comparing solvent accessibility of the pilin residues in monomer versus in filaments) yielded a similar architecture for Tfpb from *V. cholerae* (Li *et al.*, 2008; Li, Egelman and Craig 2012). In this model, consecutive subunits in a 88 Å diameter right-handed 1-start helix are separated by a rise of 8.4 Å and show 97º azimuthal rotation. Unlike in the Tfpa model in which they are exposed on the surface, the αβ-loops are implicated in holding the subunits together and are buried within the filament, whereas a sizeable segment of α1N is curiously more exposed to the solvent (Li *et al.*, 2008). This looser packing might explain, at least in part, the reduced resistance of Tfpb to denaturing agents when compared to Tfpa (Li, Egelman and Craig 2012).

A different approach has been used to model the T2SS hyper-pseudopilus of *K. oxytoca* (Campos *et al.*, 2010). In this computational approach, a multistage minimization and molecular dynamics modelling procedure used the structure of the PulG subunit and symmetry parameters of the helix obtained from classical EM studies (Campos, Francetic and Nilges 2011). This procedure allows the protomers to ‘explore’ conformations that would be missed in the above ‘rigid’ approaches. It led to one major model that closely resembles that of *N. gonorrhoeae* Tfpa, i.e. a right-handed 1-start helix with an axial rise of 10.4 Å between consecutive subunits. The salt bridges and hydrophobic contacts predicted between α1N-helices have been elegantly validated by single and complementary charge inversions and/or double Cys substitutions and cross-linking (Campos *et al.*, 2010). Using this strategy, reliable models consistent with the above ones could also be obtained for Tfpa and Tfpb (Campos, Francetic and Nilges 2011). Interestingly, in this flexible modelling approach, the predicted salt bridge between Glu_5_ (P) and Phe_1_ (P+1) in Tfpa (Craig *et al.*, 2006) and Tfpb (Li, Egelman and Craig 2012) was found only in a minority of models, since in the most stable conformation charged residues in α1C of P formed a salt bridge with Glu_5_ from P+3. This suggested that several conformational states might coexist, which is supported by EM in which a continuum of structures with different azimuthal rotations between subunits could be observed (Nivaskumar *et al.*, 2014). It was proposed that three main conformational groups might be consecutive transitions during filament assembly, which led to a model for hyper-pseudopilus assembly. In this model, a rotation-driven mechanism was proposed (Nivaskumar *et al.*, 2014) in which filament assembly is initiated by P−P+1 contacts between pilins still localized in the membrane (docking step). Then, the force generated by the traffic ATPase spools a newly docked protomer into the fibre, leading to its partial extraction from the membrane and its rotation by an average 84º. Two subsequent rounds of elongation/rotation lead to full extraction of this protomer from the membrane, and the overall 252º rotation allows P−P+3 contacts that consolidate the filament. The finding that archaella are rotating Tfp (Shahapure *et al.*, 2014) gives further support to this rotation-coupled assembly as a common mechanism for all Tff (although angles will be different in different systems).

## MOLECULAR MECHANISM OF TFF BIOGENESIS

Molecular mechanisms of Tff biogenesis are still poorly understood and we cannot definitely answer the fundamental question: How are Tff assembled? However, much progress has been made with the identification of several discrete steps in Tff biogenesis, much better knowledge of the different protein sub-complexes involved (Fig. 2) and (at least partial) 3D structural information for all the key players. Important parallels between different systems can now be drawn, but it would be imprudent to make generalized conclusions at this stage.

### Prepilin transport and processing

The translocation of prepilins across the cytoplasmic membrane and subsequent processing by the prepilin peptidase is the first and best understood stage of Tff biogenesis. As shown in *E. coli* in the absence of any other component of the Tff machinery (Strom and Lory 1987; Dupuy *et al.*, 1991), the conserved N-terminal motif of prepilins is sufficient to promote their insertion in the cytoplasmic membrane. Membrane topology is determined by the conserved sequence features of the class III signal peptide. Following the ‘positive-inside’ rule (von Heijne and Gavel 1988), the hydrophilic leader peptide remains in the cytoplasm, the hydrophobic α1N-helix behaves as a transmembrane domain, while the rest of the prepilin is exposed to the periplasmic space (Strom and Lory 1987; Dupuy *et al.*, 1991). Insertion of prepilins in the membrane relies on a universal machinery composed of the signal recognition particle (SRP) and the Sec translocon (Arts *et al.*, 2007b; Francetic *et al.*, 2007). The SRP binds the conserved N-terminal motif when the nascent prepilin polypeptide emerges from the ribosome and feeds it to the Sec translocon, which translocates it across the cytoplasmic membrane and integrates it into this lipid bilayer where it adopts its typical 3D structure.

Since class III signal peptides lack the specific cleavage residues found after the hydrophobic tract in class I and class II signal peptides (Pugsley 1993b), prepilins are catalytically processed by dedicated prepilin peptidases (Kaufman, Seyer and Taylor 1991; Nunn and Lory 1991), which in many (but not all) bacteria also often methylate the first residue of mature pilins (Strom, Nunn and Lory 1993). Prepilin peptidases form a new superfamily of polytopic membrane aspartic acid proteases lacking the canonical cleavage site and low pH requirements of classical aspartate proteases (LaPointe and Taylor 2000). Cleavage of the leader peptide occurs after the conserved Gly on the cytoplasmic side of the membrane (Fig. 2) and effectively leaves the mature pilin as a membrane protein with no remaining domain in the cytoplasm (Lemkul and Bevan 2011). Prepilin processing and N-methylation by the prepilin peptidase do not require any other component of the Tff biogenesis machinery, since they can be observed upon co-synthesis of these two proteins in a cell-free translation system (Aly *et al.*, 2013). It is very likely (although it remains to be formally demonstrated) that the IPR010627 domain catalyzes the N-methylation activity since zinc-binding via a CysXXCys-X_n_-CysXXCys motif was found to be important (Aly *et al.*, 2013). It remains to be determined whether IPR009655 in archaeal prepilin peptidases catalyzes an alternative N-terminal modification, perhaps the cyclization into a lactam ring of the N-terminal-free amino group as identified in *M. maripaludis* pilin (Ng *et al.*, 2011). Importantly, although there are exceptions (de Bentzmann *et al.*, 2006; Szabó *et al.*, 2007), many prepilin peptidases show substrate promiscuity and can process proteins harbouring (even non-canonical) class III signal peptides (Winther-Larsen *et al.*, 2005) from other Tff systems in the studied species (Nunn and Lory 1993), or even from pilins coming from other species (Nunn and Lory 1991).

Many site-directed mutagenesis studies have highlighted the importance of two conserved Asp residues in prepilin peptidases for efficient prepilin processing (LaPointe and Taylor 2000; Bardy and Jarrell 2003; Akahane *et al.*, 2005; Tomich, Fine and Figurski 2006). Critically, the recent crystal structure of the *M. maripaludis* FlaK prepilin peptidase (Hu *et al.*, 2011) provided a framework for understanding the mechanism of catalysis. FlaK, which is ‘tilted’ in the membrane, consists of a membrane-spanning central domain composed of six transmembrane helices and a soluble domain with four anti-parallel β-strands protruding into the cytoplasm, which constitutes the bulk of the archaea-specific IPR009655 motif. Strikingly, the crystal structure indicated that FlaK must undergo significant conformational change to bring the two catalytic Asp residues—separated by as much as 12 Å—close enough for catalysis to occur. This change in conformation is likely to occur upon loading of the prepilin substrate. Accordingly, interfering with this conformational shift abolished the peptidase proteolytic activity (Hu *et al.*, 2011).

Site-directed mutagenesis studies have also been performed on prepilins and revealed common principles (Strom and Lory 1991; Pugsley 1993a; Horiuchi and Komano 1998; Thomas, Chao and Jarrell 2001). The leader peptide is necessary for efficient processing—but it can in some systems be shortened without adverse effects (Horiuchi and Komano 1998; Ng *et al.*, 2009)—and the last residue (the conserved Gly) is absolutely critical. In contrast, the conserved Glu_5_ in the hydrophobic stretch of bacterial (pseudo)pilins, which appears to be important for methylation in Tfp (Pasloske and Paranchych 1988; Strom and Lory 1991) but not in T2SS (Pugsley 1993a), is always dispensable for processing. Glu_5_ pilin mutants cannot be assembled into homopolymeric Tfp (Pasloske, Scraba and Paranchych 1989; Strom and Lory 1991; Horiuchi and Komano 1998), but they can be efficiently incorporated together with wild-type subunits into compound filaments (Pasloske, Scraba and Paranchych 1989; Aas *et al.*, 2007). The lack of homopolymerization of Glu_5_ mutants is unlikely to be due to their lack of methylation since other mutants that are not methylated can be efficiently assembled into functional Tfp (Strom and Lory 1991). Therefore, the functional role of N-terminal methylation of some bacterial pilins remains enigmatic.

### Traffic ATPases power Tff (dis)assembly

Once they have been processed, mature pilins remain membrane proteins (Strom and Lory 1987; Dupuy *et al.*, 1991) and must be actively extruded from the lipid bilayer in order to be polymerized into the base of growing filaments. This process is powered by cytoplasmic proteins from the superfamily of traffic ATPases (Planet *et al.*, 2001; Peabody *et al.*, 2003), which are thought to invariably function as oligomers (Sakai, Horiuchi and Komano 2001; Rose *et al.*, 2011). These are associated with the membrane in a non-covalent fashion, via interaction with the membrane proteins involved in Tff assembly (Fig. 2) (Sandkvist *et al.*, 1995; Tripathi and Taylor 2007). Oligomerization occurs once the traffic ATPase binds ATP, which leads to its association with a membrane partner and stimulates ATPase activity (Shiue *et al.*, 2006). Consistent with membrane localization of this complex, ATPase activity is dramatically increased in the presence of phospholipids (Camberg *et al.*, 2007). In Tfpa, the extension ATPase PilF also interacts strongly with the cytoplasmic PilZ protein (Guzzo *et al.*, 2009; Georgiadou *et al.*, 2012), but the functional significance of this is unclear (Alm *et al.*, 1996a; Brown *et al.*, 2010). As shown by site-directed mutagenesis, the highly conserved Walker motifs in traffic ATPases are essential for their enzymatic activity (Turner *et al.*, 1993; Possot and Pugsley 1994; Jakovljevic *et al.*, 2008; Patrick *et al.*, 2011). These properties are shared by the retraction ATPase PilT (Brossay *et al.*, 1994; Herdendorf, McCaslin and Forest 2002; Chiang, Habash and Burrows 2005). PilT powers disassembly of pilin subunits from the base of filaments, which form a pool in the cytoplasmic membrane ready to be polymerized again (Morand *et al.*, 2004). These different traffic ATPases have distinctive sequence features which probably account for their different functional roles. For example, the C-terminal domain (CTD) of PilT contains a helical AIRNLIRE motif, which is critical for pilus retraction but is not required for ATPase activity or oligomerization (Aukema *et al.*, 2005). Two-hybrid studies in *N. meningitidis* have recently identified an intricate interaction network between the four-traffic ATPases found in this species (Georgiadou *et al.*, 2012). This introduced the notion that the situation might be more complex than alternative switching of two steadfast antagonistic motors at the base of the Tff, and that hetero-hexamers or higher order complex motors might exist. Therefore, the highly dynamic nature of Tfpa would be under the dependence of the composition of these ‘hybrid’ motors, which remains to be formally demonstrated.

Several crystallographic studies of extension motors (Yamagata and Tainer 2007; Lu *et al.*, 2013), the PilT retraction motor (Satyshur *et al.*, 2007; Misic, Satyshur and Forest 2010) and the archaellum FlaI ATPase (Reindl *et al.*, 2013), have provided insight into how chemical energy resulting from ATP hydrolysis might be transformed into mechanical energy. All of these structures describe a hexameric ring arrangement of bilobed monomers, in which distinct NTD and CTD are connected by flexible linkers. In each case, the NTD adopts a PAS-like fold usually comprising six anti-parallel β-strands flanked on one side by two or three α-helices. The CTD is topologically similar to RecA-like proteins with four signature ATPase motifs (Walker A and B motifs, and Asp and His boxes) which surround the nucleotide-binding pocket and form the catalytic centre. A striking feature of these hexameric rings is their dynamic nature, as they undergo dramatic domain movements and consequently adopt very different conformations upon ATP binding and hydrolysis. In brief, binding of the ATP involving conserved Arg residues forming a ‘basic clamp’ brings NTD and CTD from one subunit closer together (a ‘closure’ stage later released upon ATP hydrolysis), driving a ‘swinging’ motion of the neighbouring subunit CTD with some residues moving tens of Å (Satyshur *et al.*, 2007; Reindl *et al.*, 2013).

### Assembling Tff: the last frontier

How the mechanical energy generated by domain motion within traffic ATPases is translated to pilins during Tff (dis)assembly remains the main mystery in the field. Since mature pilins do not have a cytoplasmic domain (Lemkul and Bevan 2011) and Tff assembly occurs on the periplasmic side of the membrane, this mechanical energy must be transduced to pilins across the membrane, via one or several membrane proteins that together form a Tff assembly sub-complex (Fig. 2). Although there is widespread agreement on this point, discrepant findings have been reported and two different models coexist.

A seminal finding concerning this step comes from Tfpa studies showing that not all proteins essential for pilus biogenesis are involved in filament assembly *per se*. This was first established in *N. gonorrhoeae* by showing that the piliation defect in a *pilC* mutant could be suppressed by a second mutation in *pilT* (Wolfgang *et al.*, 1998b). PilC is thus dispensable for Tfp assembly in the absence of pilus retraction, suggesting that PilC exerts its role in Tfp biogenesis at a late stage (see below). Importantly, piliation is also restored in a *pilY1/T* double mutant in the phylogenetically distant *P. aeruginosa*, where PilY1 is the orthologue of meningococcal PilC (Heiniger *et al.*, 2010). An important caveat is that when piliation is not restored in a double mutant, this is (indirect) evidence that the corresponding protein might be involved in pilus assembly. This approach has been used in a systematic fashion in *N. meningitidis* and revealed that, out of the 15 proteins required for piliation, a surprisingly small number might be involved in filament assembly (PilD, PilE, PilF, PilM, PilN, PilO and PilP) since piliation could be restored in all the other double mutants (Carbonnelle *et al.*, 2006). Many of these findings are consistent with those in the closely related *N. gonorrhoeae* (Wolfgang *et al.*, 2000; Winther-Larsen *et al.*, 2005).

Therefore, since the roles of PilD, PilE and PilF are known, these results suggested that it is the PilMNOP sub-complex that uses the energy generated by PilF in the cytoplasm and translocates it across the membrane to mature pilins while polymerizing them into helical filaments (Fig. 2). Although they share little, if any, sequence homology, structural data have clearly shown that structural homologues of PilMNOP are found in other well-studied Tff systems (Korotkov, Sandkvist and Hol 2012). The structure of PilM (Karuppiah and Derrick 2011) is similar to the cytoplasmic domains of BfpC and GspL that are involved in Tfpb biogenesis and T2SS, respectively (Abendroth *et al.*, 2004a; Yamagata *et al.*, 2012). The structures of PilN and PilO (Sampaleanu *et al.*, 2009; Karuppiah *et al.*, 2013), which exhibit similar circular permutations of the ferrredoxin fold, are similar to the periplasmic domain of GspL and to GspM, respectively (Abendroth *et al.*, 2004b; Abendroth, Kreger and Hol 2009a). Finally, PilP is a structural homologue of the homology region (HR) domain that is found in all GspC proteins (Golovanov *et al.*, 2006; Korotkov *et al.*, 2011b; Tammam *et al.*, 2011; Gu *et al.*, 2012). There is now a wealth of functional information confirming the existence of a PilMNOP sub-complex at the cytoplasmic membrane. This was obtained in different bacterial species and systems, using a wide variety of approaches (stability assays in which the absence of one of these proteins negatively impacts the stability of the others, two-hybrid studies, co-immunoprecipitation, co-purification and/or co-crystallization). In brief, PilM interacts with the short cytoplasmic portion of PilN (Karuppiah and Derrick 2011; Georgiadou *et al.*, 2012), making the PilMN complex an orthologue of GspL (Fig. 2). PilN interacts with PilO (Ayers *et al.*, 2009; Sampaleanu *et al.*, 2009; Georgiadou *et al.*, 2012), which is also well documented for their GspL and GspM counterparts (Sandkvist *et al.*, 1999; Py, Loiseau and Barras 2001), forming together a PilMNO complex (Karuppiah *et al.*, 2013). PilP is also part of this complex, but it is unclear whether it interacts with PilN, PilO or both (Georgiadou *et al.*, 2012; Tammam *et al.*, 2013). Critically, consistent with a role in Tff assembly, this complex interacts with the pilin substrate and the traffic ATPase. Interaction with pilin subunits (Karuppiah *et al.*, 2013; Tammam *et al.*, 2013) occurs either via PilN (or the equivalent periplasmic portion of GspL), PilO, or both (Gray *et al.*, 2011; Georgiadou *et al.*, 2012). There is extensive experimental evidence in T2SS that the traffic ATPase GspE interacts with GspL (Sandkvist *et al.*, 1995; Py, Loiseau and Barras 1999; Possot *et al.*, 2000; Robert, Filloux and Michel 2005; Shiue *et al.*, 2006), including the 3D structure of a complex between the cytoplasmic domain of GspL (GspL_cyto_) and an N-terminal fragment of GspE (Abendroth *et al.*, 2005). In the absence of GspL, GspE mislocalizes to the cytoplasm (Sandkvist *et al.*, 1995), which is a strong indication that GspL anchors traffic ATPases to the membrane where they power Tff (dis)assembly. The very recent crystal structure of the full length GspE–GspL_cyto_ complex (Lu, Korotkov and Hol 2014b) identified linear ‘arrays’ of GspL_cyto_, which could be the driving force for the formation of the assembly sub-complex. The subsequent swinging motion of traffic ATPase domains within a hexamer would thus ‘drag’ interacting GspL together with GspM, thereby powering pilin assembly into Tff. The possibility that PilM links PilF (and PilT) with the rest of the (dis)assembly sub-complex remains to be formally demonstrated, but it is very likely since the patch of residues that mediate the interaction of GspL with GspE is conserved in PilM (Abendroth *et al.*, 2005; Karuppiah and Derrick 2011). Importantly, this might also be the norm in archaea as suggested by the finding that FlaH (that is an ATP-binding protein like PilM) interacts with the FlaI traffic ATPase (Banerjee *et al.*, 2013).

Recently, however, when the above *pilT* suppressor assay was used in *P. aeruginosa*, important discrepancies with the above model emerged. It was reported that *pilM/T*, *pilN/T*, *pilO/T* and *pilP/T* mutants exhibit piliation (although at low levels when compared to the wild type), while the *pilC/T* remains non-piliated (PilC is the orthologue of meningococcal PilG) (Takhar *et al.*, 2013). In contrast, the *pilG/T* meningococcal mutant is heavily piliated and even exhibits a partial restoration of Tfp-linked phenotypes (Carbonnelle *et al.*, 2006). The main experimental difference between the two studies is that piliation was assessed by immunofluorescence microscopy in *N. meningitidis*, which is a direct assay of Tfp production, while in the *P. aeruginosa* study it was assessed indirectly via immunodetection of the pilin subunit in sheared supernatant fractions. Their results led Takhar *et al.*, to propose that the energy generated by the traffic ATPase in the cytoplasm is translocated to pilins mainly through the PilG ‘platform’ protein, and that owing to the interaction between PilP and the secretin (see below) PilMNOP would represent a ‘secretin dynamic-associated’ sub-complex (Ayers, Howell and Burrows 2010) or a ‘connecting module’ between inner and outer membrane sub-complexes (Nivaskumar and Francetic 2014). A strong indication against such a merely connecting role is the finding that PilM, PilN and PilO orthologues are also found in Gram-positive bacteria expressing Tfp (Melville and Craig 2013). Nevertheless, the belief that PilG must play a key role in Tff assembly is longstanding (Hobbs and Mattick 1993) and mainly derives from the fact that it is universal in Tff systems (Fig. 4). Unfortunately, although PilG has been one of the first Tff proteins to be identified (Nunn, Bergman and Lory 1990), its exact function remains unknown. What is clear is that PilG is a polytopic cytoplasmic membrane protein with three transmembrane domains leaving the N-terminus and a large loop in the distal part of the protein exposed in the cytoplasm, while only a very small loop and few residues of the C-terminus are located in the periplasm (Thomas, Reeves and Salmond 1997; Arts *et al.*, 2007a). A different topology has also been reported but was judged to be uncommon (Blank and Donnenberg 2001). 3D structures of the N-terminal cytoplasmic portion of this protein (Abendroth *et al.*, 2009b; Karuppiah *et al.*, 2010; Kolappan and Craig 2013), which revealed a six-helix bundle, strongly suggested that it functions by interacting with other cytoplasmic proteins but offered little clue to its exact functional role. Accordingly, and although it is much less extensive than above, there is evidence that PilG interacts with the traffic ATPase (Py, Loiseau and Barras 2001; Chiang, Habash and Burrows 2005; Arts *et al.*, 2007a; Takhar *et al.*, 2013), with the pilin subunit (Georgiadou *et al.*, 2012), and also the above PilMNOP sub-complex (Py, Loiseau and Barras 2001; Georgiadou *et al.*, 2012), all of which are compatible with a role in Tff assembly.

Surmising that Tfp assembly is even less likely to be different in two Tfpa-producing species such as *N. meningitidis* and *P. aeruginosa* that have essentially identical sets of Tfp biogenesis proteins, than it is in different Tff systems, more direct evidence will be necessary to confirm and/or refute one, the other or both of the above models. Furthermore, how the corresponding sub-complex might then polymerize mature pilins into Tff remains to be determined.

### Crossing the outer membrane

In Gram-negative bacteria, there is an additional sub-complex in the outer membrane centred on the secretin PilQ, which forms a gated pore allowing translocation across this second permeability barrier of the Tff themselves as in the case of Tfp, or Tff-secreted substrates as in T2SS (Fig. 2). It is the finding in pathogenic *Neisseria* species that filaments in a *pilQ/T* mutant remain trapped in the periplasm that provided the most compelling evidence that Tfp emerge on the surface through the secretin pore and that this sub-complex is not involved in pilus assembly *per se* (Wolfgang *et al.*, 2000; Carbonnelle *et al.*, 2006). Secretins are a vast group of outer membrane proteins found in different bacterial secretion systems (Tff, T3SS and filamentous phages), and have therefore been intensively studied (Korotkov, Gonen and Hol 2011a). They generally share a high level of homology at their C-terminus, which is necessary for oligomerization within the outer membrane to generate multimers of usually 12–14 subunits, often heat- and SDS-resistant (Kazmierczak *et al.*, 1994; Hardie, Lory and Pugsley 1996a; Drake, Sandstedt and Koomey 1997). Towards their N-terminus there is decreasing homology (Korotkov, Gonen and Hol 2011a; Berry *et al.*, 2012). This part of the protein is constituted by a number of discrete and more flexible globular domains, which extend deeply into the periplasm, possibly up to the cytoplasmic membrane. The other member of the secretin sub-complex in most systems is PilP/GspC (Fig. 2), an inner membrane lipoprotein/bitopic protein, that interacts with the secretin and connects the inner and outer membrane sub-complexes (Possot, Gerard-Vincent and Pugsley 1999; Balasingham *et al.*, 2007; Tammam *et al.*, 2013). As revealed by their very similar 3D structures (Golovanov *et al.*, 2006; Tammam *et al.*, 2011; Gu *et al.*, 2012), PilP and the HR domain of GspC are functionally equivalent with a long disordered N-terminal ‘arm’ and a folded C-terminal globular β-sandwich domain. The globular domain of PilP interacts with the N0 domain of the secretin, thus bridging the different sub-complexes involved in Tff biogenesis (Korotkov *et al.*, 2011b; Berry *et al.*, 2012; Tammam *et al.*, 2013). An ‘outside-in’ assembly model, whereby the secretin sub-complex would form first and then recruit and stabilize the other sub-complexes, has been proposed in some species (Lybarger *et al.*, 2009; Friedrich, Bulyha and Sogaard-Andersen 2014). However, it is unclear whether this is a general feature since in *Neisseria* species intra-periplasmic Tfp can be assembled in a *pilQ/T* double mutant (Wolfgang *et al.*, 2000; Carbonnelle *et al.*, 2006), suggesting that the other sub-complexes are assembled and functional in the absence of the secretin sub-complex. Another important observation consistent with the role of the PilPQ sub-complex is the absence of both proteins in Gram-positive species expressing Tfp (Melville and Craig 2013). This suggests that PilP is unlikely to play a role in Tff assembly, which was inferred from the absence of Tfp in a meningococcal *PilP/T* mutant (Carbonnelle *et al.*, 2006). It is possible that the lack of piliation in this mutant was merely a consequence of the dramatic instability of PilN and PilO in the absence of PilP (Ayers *et al.*, 2009; Georgiadou *et al.*, 2012).

There is an abundance of structural information concerning secretins that has outlined common features. Early visualization of PilQ multimers by EM revealed ring-like dodecameric structures (Linderoth, Simon and Russel 1997; Nouwen *et al.*, 1999). Increasingly high-resolution 3D cryo-EM reconstructions showed cylindrical dodecamers spanning the periplasm consisting of a series of rings defining outer membrane and periplasmic domains with a closed central cavity (the so-called ‘periplasmic vestibule’), the size of which is compatible with the passage of Tff (Opalka *et al.*, 2003; Collins *et al.*, 2004; Chami *et al.*, 2005; Burkhardt *et al.*, 2012; Tosi *et al.*, 2014). It has been demonstrated *in vitro* that *N. meningitidis* PilQ multimers are able to physically accommodate purified Tfp while undergoing important conformational changes (Collins *et al.*, 2005). Atomic resolution 3D structure of a full-length secretin is still elusive, but structures for several of the globular domains of the periplasmic vestibule have been obtained. The N0 and N1 domains that are found in all secretins form compact and globular α/β folds (Korotkov *et al.*, 2009; Berry *et al.*, 2012). An important and unique feature in most Tfp-dependent secretins (*T. thermophilus* appears to be an exception) is the presence of one or two β-sandwich domains (B1/2) at their extreme N-terminus (Berry *et al.*, 2012).

The secretin sub-complex often contains (sometimes transiently) other components that are necessary for the pore function and/or stability (Koo, Burrows and Howell 2012). Following the seminal studies with GspS from *K. oxytoca* (Hardie, Lory and Pugsley 1996a), most of these proteins have been (mis)named pilotins. GspS, which is a small outer membrane lipoprotein (D’Enfert and Pugsley 1989), binds the CTD of GspD monomers (Daefler *et al.*, 1997), protects them from degradation and is necessary for their correct localization in the outer membrane (Hardie, Lory and Pugsley 1996a; Hardie *et al.*, 1996b). In the absence of GspS, or its mislocalization, GspD multimers mislocalize to the cytoplasmic membrane (Guilvout *et al.*, 2006). These findings, which were confirmed in many different species/systems (Shevchik, Robert-Baudouy and Condemine 1997; Schuch and Maurelli 2001), showed that the secretin does not require GspS for multimerization or membrane insertion, but rather that GspS is a chaperone that ‘pilots’ the secretin monomers to the outer membrane, which it reaches via the Lol pathway (hence its pilotin moniker). Strikingly, pilotins in different secretion systems are extremely diverse, showing no sequence or structural homology (Lario *et al.*, 2005; Tosi *et al.*, 2011). Many, possibly most, Tff systems do not need pilotins either because secretins are lipoproteins themselves (Schmidt *et al.*, 2001; Viarre *et al.*, 2009) or use general pathways for protein targeting to the outer membrane (Voulhoux *et al.*, 2003). Another protein that is often part of secretin sub-complexes is PilW (Fig. 2). This outer membrane lipoprotein (Carbonnelle *et al.*, 2005), which is almost exclusively found in Proteobacteria and most likely restricted to Tfpa (Fig. 4), has been found to interact with PilQ (Koo *et al.*, 2013) and is key for the stability of PilQ multimers. Indeed, in PilW absence PilQ multimers could not be detected (Carbonnelle *et al.*, 2005; Nudleman, Wall and Kaiser 2006; Koo *et al.*, 2008). Unlike what has been observed for GspS (Hardie *et al.*, 1996b), PilW mislocalization does not prevent PilQ localization to the outer membrane and/or piliation (Koo *et al.*, 2008; Szeto *et al.*, 2011) and it is therefore not a *bona fide* pilotin. Two PG-binding proteins, FimV and TsaP, have also been found as part of the secretin sub-complex in some Tfpa-expressing species, but it is unclear if this is conserved in other Tff. While FimV has been shown in *P. aeruginosa* to be important for PilQ stability and Tfp-linked functions (Wehbi *et al.*, 2011), TsaP was identified as a protein interacting peripherally with secretin rings in *N. gonorrhoeae* (Siewering *et al.*, 2014). Although lack of TsaP did not affect PilQ multimerization, the secretin channel was apparently inactive since a *tsaP* mutant exhibited intra-periplasmic fibres, reminiscent of those in a *pilQ/T* mutant (Wolfgang *et al.*, 2000; Carbonnelle *et al.*, 2006). These PG-binding proteins were suggested to anchor the secretin multimers to the cell wall, enabling them to withstand dramatic forces generated during pilus (dis)assembly, which warrants further investigation.

### Late stages in Tff biogenesis

As mentioned above, most proteins essential for Tfpa biogenesis are actually dispensable for filament assembly *per se* since the piliation defect in the corresponding genes could be suppressed by a second mutation in *pilT*. Except for *pilQ/T*, all the other double mutants display surface-exposed Tfp (Carbonnelle *et al.*, 2006), suggesting that the corresponding proteins (PilC, PilG, PilH, PilI, PilJ, PilK and PilW) act later than PilQ. Their putative role is to shift the Tfp dynamics towards assembly and recent publications have shed light on the function of the pilin-like proteins (PilH, PilI, PilJ and PilK) and PilC/PilY1.

In each Tff system, there are almost invariably additional genes beside the major (pseudo)pilin that encode proteins with class III signal peptides, are cleaved by the prepilin peptidase and play key roles in Tff biology. PilH, PilI, PilJ and PilK are thus required for Tfpa biogenesis (Winther-Larsen *et al.*, 2005; Carbonnelle *et al.*, 2006), and their GspH, GspI, GspJ and GspK counterparts are essential for T2SS (Lu, Motley and Lory 1997). An idea of the role of these minor (pseudo)pilins came from the 3D structure of a GspIJK hetero-trimer that revealed a Tff-like architecture with an axial rise of 10 Å between neighbouring subunits (Korotkov and Hol 2008). The fourth pseudopilin GspH was found to bind to GspJ at the base of this trimer (Douzi *et al.*, 2009) and the T2SS substrate was found to interact with its tip (Douzi *et al.*, 2011). Importantly, GspK that caps this complex has a large domain positioned in such a way that no additional subunit can be added above it (Korotkov and Hol 2008), which would be consistent with a localization at the tip of pseudopili and a possible role in priming filament assembly. However, in *P. aeruginosa* Tfp, the corresponding proteins have not been detected at the tip but rather distributed throughout the filaments (Giltner, Habash and Burrows 2010). Nevertheless, in accord with this scenario, hyper-pseudopilus abundance and/or length was found to be affected in mutants in *gspI*, *gspJ* and *gspK* (but not *gspH*) and was abolished in the concurrent absence of all these proteins (Durand *et al.*, 2005; Cisneros *et al.*, 2012a). Cysteine cross-linking experiments and molecular dynamics simulations outlined a model in which GspI and GspJ form a staggered complex which recruits GspK, partially extracting it from the membrane by 10 Å (Cisneros *et al.*, 2012a). This was proposed to ‘kickstart’ initiation of pseudopilus assembly, possibly by activating the assembly ATPase in the cytoplasm (although this remains purely speculative). The finding that the *E. coli* Tfp equivalents of these pseudopilins (from the PpdD pilus) can complement the hyper-pseudopiliation defect in a *gspHIJK* polymutant is strong evidence that the role of these proteins in Tff assembly might be conserved (Cisneros *et al.*, 2012b). In Tfp, however, the finding in *N. gonorrhoeae* that wild-type levels of piliation can be restored in the concurrent absence of PilH, PilI, PilJ and PilK when *pilT* is also mutated (Winther-Larsen *et al.*, 2005) seems difficult to reconcile with a role of these proteins in initiating filament assembly.

PilC/PilY1 is a protein found predominantly in Tfpa produced by Proteobacteria (Fig. 4), first identified in *N. gonorrhoeae* (Jonsson, Nyberg and Normark 1991). Orthologues in different species have diverse NTD, while their C-terminus is highly conserved. The protein is predicted to be associated with the outer membrane (Rahman *et al.*, 1997; Carbonnelle *et al.*, 2005), although a pilus localization has also been proposed (Rudel, Scheuerpflug and Meyer 1995). A series of studies in different species have shown that PilC is a bifunctional protein involved in Tfp-mediated adhesion (already discussed) and pilus biogenesis. The conserved CTD is responsible for PilC role in Tfp biogenesis (Orans *et al.*, 2010). The 3D structure of the corresponding domain in PilY1 revealed a modified β-propeller fold with a distinct and highly conserved EF-hand-like calcium-binding site (Orans *et al.*, 2010). Calcium was proposed to control PilT-mediated pilus retraction, consistent with the phenotype of a *pilC/T* mutant. Calcium-bound PilY1 inhibits PilT-mediated pilus retraction, while in a calcium-free state it is unable to do so, resulting in a non-piliated phenotype. However, although later studies agree that calcium binding by PilC is key for Tfp biology in other piliated proteobacterial species as well, no major effect on piliation was seen in calcium-free mutants (Cheng *et al.*, 2013; Porsch *et al.*, 2013).

## CONCLUDING REMARKS

Despite considerable progress, our understanding of Tff biology remains incomplete and there are important gaps in knowledge to be filled. Obviously, the main challenge consists in improving our understanding of the molecular mechanisms of Tff assembly. Perhaps the most spectacular advances in recent years came from structural studies showing that proteins in diverse Tff systems that have diverged to the point that no sequence homology is discernible do exhibit highly similar 3D structures. This is a strong argument in favour of the notion that common molecular principles govern Tff biology. Additional structural information has the potential to further improve our understanding of these principles. For example, cryo-EM combined with direct electron detectors (Lu *et al.*, 2014a) could be used to obtain much higher, possibly atomic, resolution reconstruction of various Tff, including Flp pili that are formed of a ‘minimal’ pilin (Tomich, Planet and Figurski 2007). The 3D structure of more ‘orphan’ Tff proteins from less well characterized systems might reveal that they are structural homologues of bacterial proteins to which they bear no sequence homology (e.g. is FlaH an orthologue of PilM?), fuelling the emergence of one unifying mechanism for Tff assembly. Atomic-resolution structures of a secretin channel or a bacterial prepilin peptidase in complex with a prepilin substrate would shed more light on these key steps in Tff biology. Finally, although this promises to be utterly complex, it would be worth trying to determine the structure of the various sub-complexes discussed here (full-length proteins rather than soluble domains) because this would dramatically improve our understanding of Tff biology and bring it a little closer to the exquisite understanding of pilus assembly by the chaperone–usher pathway (Phan *et al.*, 2011). Such advances will be instrumental in the rational design of specific inhibitors of Tff assembly, analogues of ‘pilicides’ interfering with pilus assembly by the chaperone–usher pathway (Pinkner *et al.*, 2006) or curli biogenesis (Cegelski *et al.*, 2009), which would represent exceptionally broad-spectrum anti-microbial compounds (Fig. 4).

Structural biology is obviously not the only research avenue that should be privileged, and further genetic, biochemical and dynamic modelling studies of different Tff properties and/or proteins are warranted. As confirmed by the wealth of interesting data coming from archaeal Tff (Jarrell *et al.*, 2013), future research should not only favour the ‘historic’ models (although there is still plenty to be learned in T2SS, Bfp, Tcp or *Neisseria* and *P. aeruginosa* Tfpa) but should be extended to new models, more distant from an evolutionary point of view. An in-depth study of Gram-positive Tfp would be for example of great interest as such bacteria are inherently ‘simpler’ because of the absence of an outer membrane (Melville and Craig 2013). This could be key in understanding how Tff cross the PG. Another attractive ‘reductionist’ approach would be to determine which proteins are necessary and sufficient for filament assembly by creating a minimal system, which could be done in several ways. Similar to what was done for Tfpb or T2SS with the transfer of the entire corresponding operons (D’Enfert, Ryter and Pugsley 1987; Sohel *et al.*, 1996; Stone *et al.*, 1996), a surrogate non-piliated organism could be used to determine which subset of genes is sufficient to promote Tff assembly. The *pilT* suppressor assay pioneered in *Neisseria* species (Wolfgang *et al.*, 1998b) could be extended by creating polymutants in a *pilT* mutant background in which all the genes not involved in Tff assembly would be deleted. Ultimately, a minimal Tff assembly system could be reconstituted from proteins expressed in a cell-free system (Aly *et al.*, 2013) or even purified, as has been achieved for type I pili (Nishiyama *et al.*, 2008). All of these studies, and many more, will undoubtedly continue to ‘feed’ the Tff field with interesting results and exciting concepts in the years to come.
